# The Molecular Complex between Staphylococcal Adhesin SpsD and Fibronectin Sustains Mechanical Forces in the Nanonewton Range

**DOI:** 10.1128/mBio.00371-20

**Published:** 2020-07-07

**Authors:** Felipe Viela, Marion Mathelié-Guinlet, Giampiero Pietrocola, Pietro Speziale, Yves F. Dufrêne

**Affiliations:** a Louvain Institute of Biomolecular Science and Technology, UCLouvain, Louvain-la-Neuve, Belgium; b Department of Molecular Medicine, Unit of Biochemistry, University of Pavia, Pavia, Italy; c Walloon Excellence in Life Sciences and Biotechnology (WELBIO), Wavre, Belgium; University of Washington

**Keywords:** single molecule, mechanostability, binding force, staphylococcal adhesion, SpsD, fibronectin, adhesion, staphylococci

## Abstract

The bacterial pathogen Staphylococcus pseudintermedius is involved in canine otitis externa and pyoderma as well as in surgical wound and urinary tract infections. Invasion of canine epithelial cells is promoted by S. pseudintermedius fibronectin (Fn)-binding proteins SpsD and SpsL through molecular interactions that are currently unknown. By means of single-molecule experiments, we discover that both adhesins have distinct molecular mechanisms for binding to Fn. We show that the SpsD-Fn interaction has a strength equivalent to that of a covalent bond (∼1.5 to 1.8 nN), which is an order of magnitude stronger than the binding force of classical receptor-ligand complexes. We suggest that this extreme mechanostability originates from the β-sheet organization of a tandem β-zipper. Upon binding to FnI modules, the intrinsically disordered binding sequences of SpsD would shift into an ordered structure by forming additional β-strands along triple peptide β-sheets in the Fn molecule. Dynamic force measurements reveal an unexpected behavior, i.e., that strong bonds are activated by mechanical tension as observed with catch bonds. By contrast, the SpsL-Fn interaction involves multiple weak bonds (∼0.2 nN) that rupture sequentially under force. Together with the recently described dock, lock, and latch complex, the ultrastrong interaction unraveled here is among the strongest noncovalent biological interaction measured to date. Our findings may find applications for the identification of inhibitory compounds to treat infections triggered by pathogens engaged in tandem β-zipper interactions.

## Abstract (precis)

Binding of Staphylococcus pseudintermedius surface proteins SpsD and SpsL to fibronectin (Fn) plays a critical role in the invasion of canine epithelial cells. Here, we discover that both adhesins have different mechanisms for binding to Fn. The force required to separate SpsD from Fn is extremely strong, consistent with the unusual β-sheet organization of a high-affinity tandem β-zipper. By contrast, unbinding of the SpsL-Fn complex involves the sequential rupture of single weak bonds. Our findings may be of biological relevance as SpsD and SpsL are likely to play complementary roles during invasion. While the SpsD β-zipper supports strong bacterial adhesion and triggers invasion, the weak SpsL interaction would favor fast detachment, enabling the pathogen to colonize new sites.

## INTRODUCTION

Staphylococcus pseudintermedius is an opportunistic pathogen that colonizes the nares and perineum of healthy dogs. Disruption of the normal skin flora, damage to the cutaneous barrier by pruritic conditions (e.g., hypersensitivities), and primary immunodeficiencies can lead to skin infections such as pyoderma caused by this organism (1). In addition, over the last 2 decades, methicillin-resistant S. pseudintermedius has emerged as a major problem in veterinary clinics worldwide (2, 3). Several episodes of life-threatening human infections by S. pseudintermedius have also been reported, mainly after contacts with dogs (4, 5).

**FIG 1:**
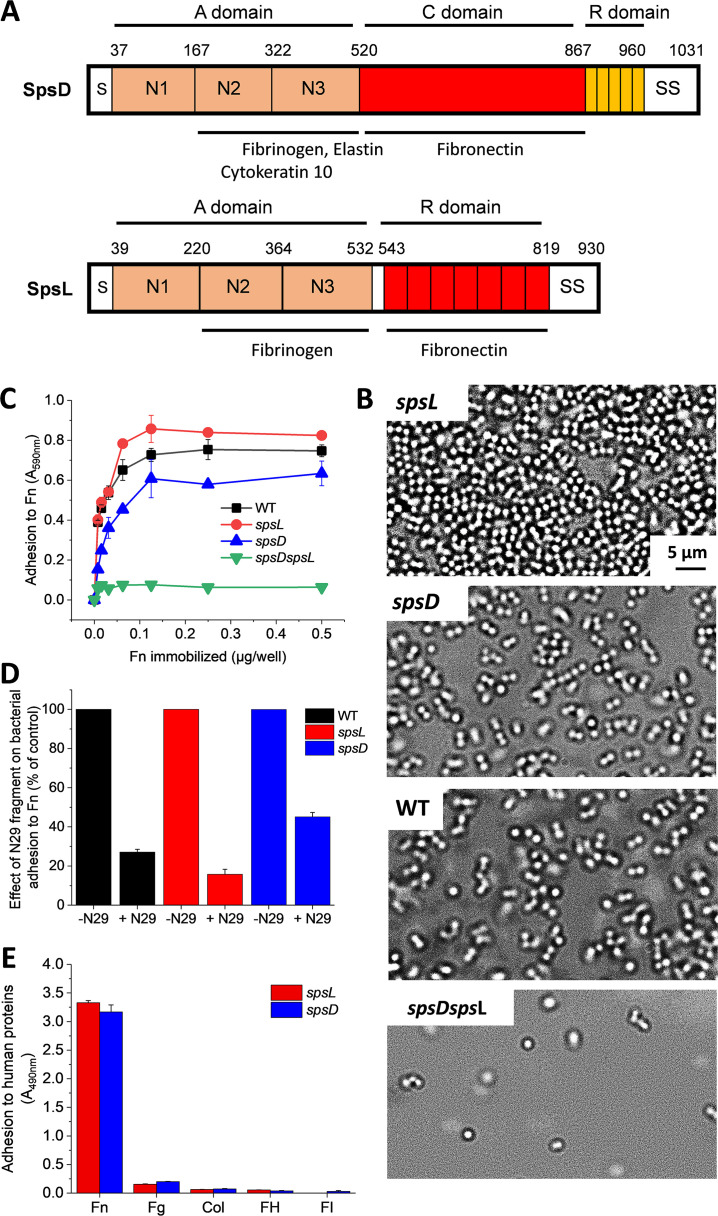
Role of Fn binding by SpsD and SpsL in S. pseudintermedius adhesion. (A) Schematic representation of SpsD and SpsL proteins from S. pseudintermedius ED99. The A domain of SpsD spans residues 37 to 519 following the signal sequence (S). This is followed by a connecting region C (residues 520 to 866) and a repeat region R. The number of repeats varies from strain to strain resulting in proteins of slightly different sizes. A sorting signal (SS) containing an LPXTG motif, a hydrophobic domain, and a positively charged stretch occurs at the extreme C terminus. SpsL includes a signal sequence (S) at the N terminus followed by an A domain (aa 39 to 531), an R domain containing seven tandem repeats (aa 543 to 818), and a C-terminal sorting signal (SS). (B) Optical microscopy images of bacteria adhering to Fn-coated surfaces, showing the critical role of SpsD and SpsL in bacterial adhesion to Fn. (C) Adhesion of bacteria to immobilized Fn. Microtiter wells coated with increasing concentrations of Fn were incubated with S. pseudintermedius ED99 WT, single-mutant cells, and double-mutant cells. After fixation with formaldehyde and staining with crystal violet, adhering cells were quantified by measuring the absorbance at 595 nm in a plate reader. Means and standard deviation of results of two independent experiments, each performed in triplicate, are presented. (D) Inhibitory effect of the N29 of Fn on S. pseudintermedius adhesion to Fn. Microtiter wells were coated with Fn and then incubated with S. pseudintermedius strain ED99 WT or its single mutants in the presence of excess amounts of N29. After several washings, the wells were stained with crystal violet and the absorbance measured as above. Adhesion of bacteria in the absence of N29 is reported as control. Means and standard deviation of results of two independent experiments, each performed in triplicate, are presented. (E) Binding of extracellular matrix and plasma proteins to SpsD_520–846_ and SpsL_538–823_ regions. Adhesin fragments (1 μg) were immobilized onto microtiter wells and then incubated with Fn, fibrinogen (Fg), collagen type I (Col), factor H (FH), and factor I (FI). Binding was measured by addition of specific antibodies to each ligand. The data are the mean values ± SD from three independent experiments.

In staphylococci, a family of cell wall-anchored surface proteins termed microbial surface components recognizing adhesive matrix molecules (MSCRAMMs) mediate bacterial adherence to extracellular matrix proteins of the host (6). Compared to Staphylococcus aureus, the interaction of S. pseudintermedius with host proteins is less characterized, but several strains have been shown to bind to fibronectin (Fn), fibrinogen, cytokeratin 10, elastin, collagen type I, vitronectin, and laminin (7, 8). A genome-wide screen revealed 18 genes encoding putative cell wall-anchored S. pseudintermedius surface proteins (9, 10). Of these, Fn-binding proteins SpsD and SpsL are believed to be important in host tissue colonization and infection (9). The primary translation product of the *spsD* gene contains 1,031 residues, has an N-terminal secretory signal sequence and a C-terminal cell wall-anchoring domain comprising an LPDTG motif, a hydrophobic transmembrane domain, and a short sequence rich in positively charged residues. The N-terminal end of SpsD consists of an A domain 40% identical to the fibrinogen-binding domain of FnBPB from S. aureus and is involved in binding to fibrinogen, cytokeratin-10, and elastin (11). This domain is followed by a connecting region, region C, which interacts with Fn, and the repeat region R (12) (Fig. 1A). SpsL is a protein of 930 residues that includes a signal sequence at the N terminus followed by a fibrinogen-binding A domain with three IgG-like folds (N1 to N3) (13), an R domain containing seven tandem repeats that confer Fn-binding capacity (12), and a C-terminal sorting signal (Fig. 1A).

In S. aureus, cellular invasion is triggered by the interaction between Fn-binding proteins FnBPA and FnBPB and the α_5_β_1_ integrin in the host cell membrane (12). The key to this process is the formation of a Fn bridge between FnBPs and integrin. Soluble Fn is made of multiples modules (<100 amino acids [aa]) called type I, II, III repeats. FnBPA features eleven nonidentical, unfolded Fn-binding repeats (FnBRs) that bind with four sequential modules of the N-terminal FI domain via a tandem β-zipper featuring an unusual β-sheet organization (14, 15).

While Fn binding by SpsL and SpsD supports invasion of canine epithelial cells by S. pseudintermedius (12), the molecular interactions involved are not known. Two important yet unsolved questions are the following. (i) What are the binding strengths of SpsD and SpsL? (ii) As the two adhesins fulfil similar functions, do they share the same binding mechanism? Using single-molecule atomic force microscopy (AFM) experiments (16, 17), we show that SpsD and SpsL are engaged in very different interactions with Fn. While SpsL and Fn form weak bonds, SpsD binds Fn via extremely strong forces, reflecting the β-sheet organization of a tandem β-zipper. These results may contribute to the development of antiadhesion approaches to treat infections caused by S. pseudintermedius and other bacterial pathogens engaged in tandem β-zipper interactions.

## RESULTS

### SpsD and SpsL favor bacterial adhesion to immobilized Fn.

We studied Fn binding using S. pseudintermedius ED99 mutant *spsL* and *spsD* strains expressing either SpsD or SpsL. As controls, we used cells expressing SpsD and SpsL (wild-type [WT] cells) and mutant cells lacking both adhesins (*spsD spsL* mutant cells). As reported previously (10), deletion of one type of adhesin does not affect expression of the other.

Using optical microscopy, we found that *spsL* mutant cells adhered strongly to Fn-coated substrates, while *spsD* mutant and WT cells showed lower levels of adhesion (Fig. 1B). Poor adhesion was observed with the double mutant, implying that SpsD and SpsL are the only Fn-binding proteins expressed at the cell surface. The same adhesion pattern was observed in an assay in which the bacteria were allowed to attach to microtiter wells coated with Fn (Fig. 1C). The higher level of adhesion of the *spsL* mutant compared to that of the WT strain could be due to a better exposure of SpsD proteins to the ligand on the bacterial surface. It is also possible that, unlike in the WT where SpsD and SpsL proteins compete with each other for Fn binding, no such competition occurs in the *spsL* mutant and Fn is only captured by SpsD.

Moreover, adhesion of cells from the WT and single-mutant strains was inhibited in the presence of the N-terminal fragment of Fn (N29), indicating that this region is involved in Fn binding by SpsD and SpsL as for FnBPA (Fig. 1D). To further evaluate the specificity of Fn binding, the active regions (based on the sequence alignment with S. aureus FnBPA) SpsD_520−846_ (C domain, connecting region) and SpsL_538−823_ (R domain, repetitive region) were immobilized onto microtiter wells and tested for their ability to bind various extracellular matrix and plasma proteins, i.e., Fn, fibrinogen, collagen type I, factor H, and factor I. Substantial binding was only observed for Fn, demonstrating that the latter interacts specifically with SpsD_520−846_ and SpsL_538−823_ (Fig. 1E).

### SpsD and SpsL bind to Fn with different affinities.

**FIG 2:**
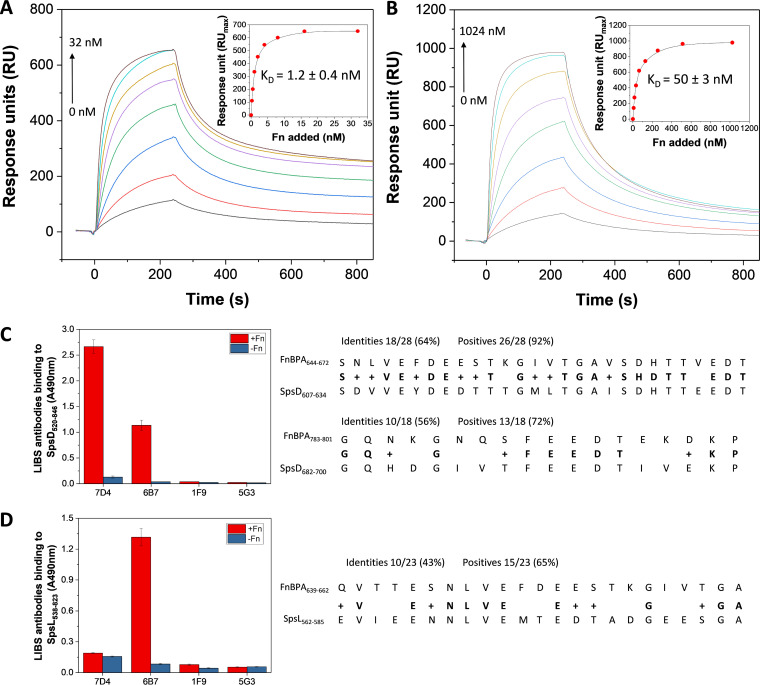
Affinity and immunological reactivity of SpsD and SpsL. (A, B) SPR analysis of the SpsD-Fn and SpsL-Fn interactions. Sensorgrams show the binding of Fn to SpsD_520–846_ (A) and SpsL_538–823_ (B) immobilized on sensor chips. Insets are plots of RU_max_ versus Fn concentrations. The data points were filled with the Langmuir equation describing a 1:1 binding model. Graphs show representative data out of three experiments. (C, D) Cross-reactivity of LIBS monoclonal antibodies with SpsD_520–846_ and SpsL_538–823_. Microtiter wells were coated with recombinant SpsD_520–846_ (C) or SpsL_538–823_ (D) and incubated with the indicated FnBPA LIBS monoclonal antibodies in the absence or presence of Fn. After washing, antibody binding was detected by incubating the wells with a rabbit anti-mouse HRP-conjugated antibody. Error bars represent SD from means of triplicate determinations. The sequences of FnBPB-5 and FnBPA-9 repeats (Swiss-Prot accession number Q53682) aligned with internal sequences of SpsD_520–846_ and SpsL_538–823_ by using the protein BLAST tool are reported on the right side of each panel.

We analyzed the interaction of Fn with SpsD_520−846_ and SpsL_538−823_ using surface plasmon resonance (SPR) by immobilizing the bacterial domains on a chip and injecting Fn in the mobile phase (Fig. 2A and B). The best fit of the data points was obtained with the Langmuir isotherm equation describing a one-site binding model. From this analysis, we obtained dissociation constant (*K_D_*) values of 1.2 ± 0.4 nM and 50 ± 3 nM for the SpsD-Fn and SpsL-Fn complexes, respectively. This shows that both adhesins strongly bind to Fn but that SpsD clearly exhibits a higher affinity than SpsL.

### SpsD and SpsL share conformational epitopes with FnBPA.

We also wondered if ligand-induced binding site (LIBS) monoclonal antibodies (MAbs) could recognize epitopes involved in Fn (or N29) binding in the SpsD_520−846_ and SpsL_538−823_ domains. We previously showed that a family of LIBS MAbs against S. aureus FnBPA recognizes specific repeats of the adhesin in the presence of Fn; specifically, MAbs 6B7 and 7D4 recognized conformational neoepitopes in the FnBPA-5 and FnBPA-9 repeats, respectively (18). We found that MAbs 6B7 and 7D4 strongly reacted with the C region of SpsD (Fig. 2C), while only 6B7 showed reactivity for the SpsL fragment (Fig. 2D). Although the connecting C region of SpsD and the repetitive region of SpsL bind to Fn, the conformational epitopes present in SpsD are different from those present in the R region of SpsL. Hence, 7D4 recognizes a specific epitope that is only formed when SpsD, but not SpsL, binds to Fn. We hypothesize that these regions may have a flexible structure that can shift from a disordered to an ordered structure in the presence of Fn, as in the FnBPA-Fn interaction. To support this, the secondary structures of the connecting region of SpsD (SpsD_520−846_) and the repetitive domain of SpsL (SpsL_538−823_) were analyzed by circular dichroism (CD) (see Fig. S1 in the supplemental material). Both proteins featured quite similar CD spectra and harbored significant unordered regions (43% random coil). The reactivity of LIBS antibodies was correlated with sequence alignments of the FnBPA-5 and FnBPA-9 repeats versus the Fn-binding regions of SpsD_520−846_ and SpsL_538−823_ (Fig. 2C and D, right). A high degree of identity/similarity was observed for SpsD as follows: 64%/92% for FnBPA-5_644−672_ versus the SpsD_607−634_ sequence and 56%/72% for FnBPA-9_783−801_ versus the SpsD_682−700_ sequence. There was also identity and similarity between FnBPA-5_639−662_ and SpsL_562−585_ (43%/65%) yet to a lower extent than that in SpsD, while FnBPA-9 showed no identity/similarity to SpsL_538−823_. So, there is a higher level of identity/similarity for SpsD than for SpsL. Together, these data suggest that S. pseudintermedius SpsD and SpsL share with S. aureus FnBPA disordered epitopes that acquire an ordered structure upon binding to Fn. As a proof of the specificity of the formed neoepitopes recognized by MAbs 6B7 and 7D4, no reactivity for SpsD and SpsL was exhibited by the MAbs 1F9 and 5G3 (Fig. 2C and D).

10.1128/mBio.00371-20.1 FIG S1 Spectroscopic analyses of SpsL_538–823_ and SpsD_520–846_ domains. Far-UV CD spectra were recorded at 10 μM concentration for both proteins in 20 mM phosphate buffer, pH 7.0. The predicted secondary structure composition of each protein is reported in the insert. The spectra are the average of 10 scans and corrected for buffer blank. Download FIG S1, DOCX file, 0.4 MB. Copyright © 2020 Viela et al. 2020 Viela et al. This content is distributed under the terms of the Creative Commons Attribution 4.0 International license.

### S. pseudintermedius engages in two modes of interaction with Fn.

**FIG 3:**
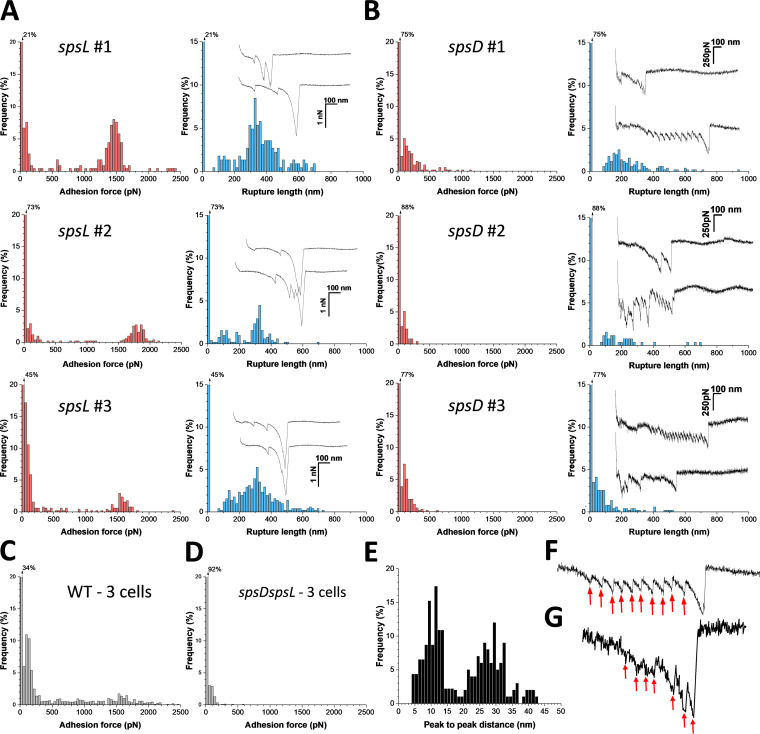
Adhesion forces between single bacteria and Fn substrates. (A, B) Maximum adhesion force (left) and rupture length (right) histograms with representative retraction force profiles (insets) obtained by recording force-distance curves in PBS between three S. pseudintermedius *spsL* mutant cells (A) or *spsD* mutant cells (B) from different cultures and Fn substrates. Force data obtained under the same conditions for three S. pseudintermedius WT cells (C) and three *spsD spsL* mutant cells (D). (E) Distribution of peak-to-peak distances associated with Fn unfolding, in the *spsD* mutant cells documenting unfolding events separated by 28 ± 5 nm (*n *= 45 curves); distance of 10 ± 2 nm (*n *= 20) are also observed, which might reflect unfolding of SpsL repeats. (F) Typical unfolding pattern of Fn; (G) sequential rupture of SpsL domains used to build the histogram shown in panel E.

The molecular interactions of SpsD and SpsL were first studied by measuring the forces between a single bacterium and Fn-coated surfaces. Figure 3A shows the adhesion forces and rupture lengths obtained for three representative *spsL* mutant cells (for more cells, see Fig. S2A in the supplemental material). Strong adhesion events were detected with mean forces of 1,444 ± 117 pN (mean ± standard deviation [SD]; *n *= 111 adhesive curves), 1,754 ± 174 pN (*n *= 85), and 1,541 ± 154 pN (*n *= 74) for cells 1, 2, and 3, respectively. These forces were specific to SpsD, as they were abolished in *spsD spsL* mutant cells (Fig. 3D). The rupture length is the distance from the contact point to the unbinding point. Most bonds ruptured around ∼300 nm, but some ruptures up to ∼700 nm were also observed. Assuming that the processed mature adhesin comprises 1,031 residues and that each amino acid contributes 0.36 nm to the contour length of the polypeptide chain, the fully extended protein should be ∼371 nm long. This suggests that both SpsD and Fn are being stretched upon pulling the cells away from the Fn surfaces.

10.1128/mBio.00371-20.2 FIG S2 Single-cell force spectroscopy of SpsD/SpsL-Fn interactions. Maximum adhesion force (left) and rupture length (right) histograms obtained by recording force-distance curves in PBS between three additional S. pseudintermedius *spsL* mutant (A) and *spsD* mutant (B) cells and Fn-coated substrates. Download FIG S2, DOCX file, 0.7 MB. Copyright © 2020 Viela et al. 2020 Viela et al. This content is distributed under the terms of the Creative Commons Attribution 4.0 International license.

The *spsD* mutant cells featured very different interactions in that strong adhesion was never observed. Instead, weak forces of 168 ± 93 pN (mean ± SD; *n *= 246 adhesive curves; 3 cells) and 219 ± 111 nm rupture lengths were observed (Fig. 3B; for more cells, see Fig. S2B). These forces were specific as their frequency was largely reduced in the double mutant (from 66% to 8%; mean from 3 cells) (Fig. 3D). WT cells featured a combination of both weak and strong forces (Fig. 3C), which is not surprising as they express both adhesins. Strikingly, *spsD* mutant cells, but not *spsL* mutant cells, featured adhesive curves (∼10% of all adhesive curves) with sawtooth patterns with successive unbinding events (7 to 15) of 141 ± 30 pN magnitude and peak-to-peak distance of 28 ± 5 nm, matching the 28-nm unfolding distance of FnIII repeats (Fig. 3E and F) (19). Therefore, rupture of the SpsL-Fn complex seems to be associated with the unfolding of multiple FnIII repeats. In SpsD, unfolding of FnIII repeats was not observed. Another set of force curves (∼5%) showed multiple peaks separated by 10 ± 2 nm, consistent with the unfolding of SpsL repeats (7 × 37 residues) (Fig. 3E and G). This fits with a bond rupture in which individual SpsL repeats unfold sequentially and detach from the Fn region. Collectively, these results show that SpsD and SpsL mediate bacterial adhesion to Fn through different interactions, i.e., single strong forces versus multiple weak forces, therefore, suggesting that distinct ligand-binding mechanisms occur.

### The SpsD-Fn interaction is extremely strong.

**FIG 4:**
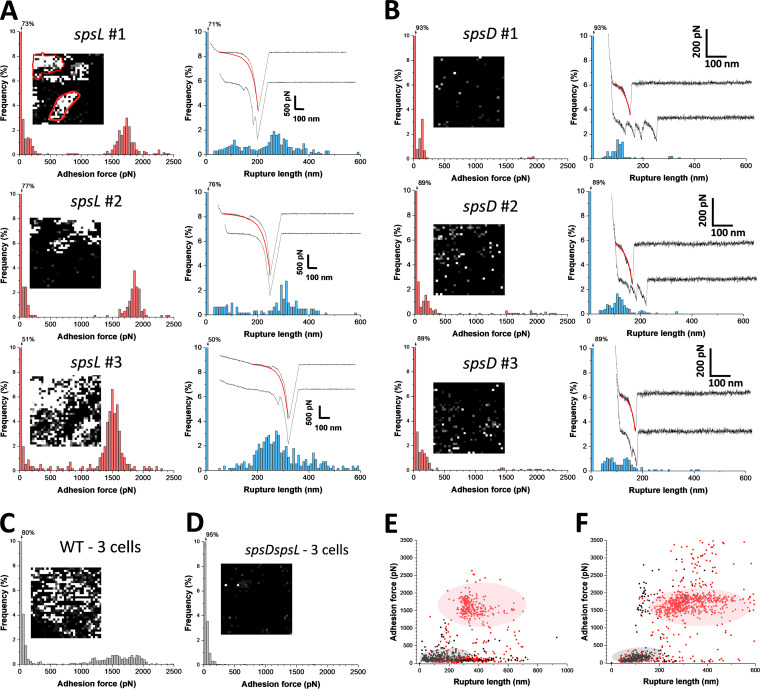
Strength of single SpsD-Fn and SpsL-Fn bonds in living bacteria. (A, B) Maximum adhesion force (left) and rupture length histograms (right) obtained by recording force-distance curves in PBS between three different S. pseudintermedius *spsL* mutant cells (A) or *spsD* mutant cells (B) and AFM tips functionalized with Fn. Insets show representative adhesion maps (size, 500 nm by 500 nm) and representative retraction force profiles along with WLC fittings (red line, root mean square [RMS] [SpsD] of ∼75 pN and RMS (SpsL) of <10 pN). (C, D) Force data obtained under the same conditions for three S. pseudintermedius WT cells (C) and three *spsD spsL* mutant cells (D). (E, F) Plots of adhesion forces versus rupture lengths for *spsL* (red) and *spsD* (black) mutant cells studied either by single-cell (*n *= 999 data points) (E) or single-molecule (*n *= 1,214 data points) (F) experiments.

To study the mechanical strength of individual bonds, single adhesins were picked up and pulled with an AFM tip modified with Fn (Fig. 4). Strong unbinding forces were measured between *spsL* mutant cells and Fn tips (Fig. 4A; see also Fig. S3A in the supplemental material), with a magnitude of ∼1,500 to 2,000 pN (cell 1, 1,502 ± 132 pN from *n *= 218 adhesive force curves; cell 2, 1,703 ± 110 pN, *n *= 167; cell 3, 1,815 ± 128 pN, *n *= 215). These forces are specific (see *spsD spsL* mutant strain data in Fig. 4D) and in the same range as those recorded on whole cells. As intermediate forces were never observed, we can exclude that strong forces originate from the rupture of a variable number of weak bonds. Protein extensions of 323 ± 92 nm (*n *= 600; 3 cells) were observed, suggesting that they reflect the extension (unfolding) of both SpsD and Fn. For *spsD* mutant cells, however, adhesion forces of only 158 ± 69 pN (mean ± SD; *n *= 204 adhesive curves; 3 cells) (Fig. 4B; see also Fig. S3B) were found together with much shorter rupture distances (111 ± 34), implying that Fn is in a globular form or only partially extended. The ∼158 pN force is close to that of whole cells (∼168 pN) and agrees well with the binding strength between Fn- and single FnBPA-binding repeats (∼200 pN [19]). Again, a number of curves showed multiple peaks separated by ∼10 nm, consistent with the unfolding of individual SpsL repeats. Force peaks were well described by the worm-like chain (WLC) model (Fig. 4B, insets and red lines) using a persistence length of 0.4 nm as follows: *F*_(_*_x_*_)_ = *k*_b_*T*/*l*_p_[0.25(1 − *x*/*L*_c_) − 2 + *x*/*L*_c_ − 0.25], where *L*_c_ and *l*_p_ are the contour length and persistence length of the molecule, *k*_b_ is the Boltzmann constant, and *T* is the absolute temperature.

10.1128/mBio.00371-20.3 FIG S3 Single-molecule force spectroscopy of SpsD/SpsL-Fn interactions. Maximum adhesion force (left) and rupture length (right) histograms obtained by recording force-distance curves in PBS between Fn functionalized tips and three additional S. pseudintermedius *spsL* mutant (A) and *spsD* mutant (B) cells. Download FIG S3, DOCX file, 0.7 MB. Copyright © 2020 Viela et al. 2020 Viela et al. This content is distributed under the terms of the Creative Commons Attribution 4.0 International license.

We believe that the forces reported herein mostly reflect single interactions since (i) force distributions were narrow and did not feature intermediate values usually associated with multiple bonds breaking simultaneously, (ii) single-cell and single-molecule experiments lead to very similar sharp distributions, and (iii) 1-ethyl-3-(3- dimethylaminopropyl)-carbodiimide (EDC)/*N*-hydroxysuccinimide (NHS) chemistry, used in this study, has been shown to favor single-molecule detection over the years, which is further supported by the occurrence of single well-defined peaks (versus multiple peaks).

Single-molecule mapping revealed that SpsD binding events were localized heterogeneously and formed nanodomains (Fig. 4A, left insets), while SpsL was randomly localized on the bacterial cell surface (Fig. 4B, left insets), suggesting a different binding mechanism. Finally, plots of the binding strengths versus rupture lengths (Fig. 4E and F) emphasize the strong similarities between single-molecule and whole-cell experiments and demonstrate the major difference between the strong, long-range SpsD interaction and the weak, short-range SpsL interaction.

### Dynamics of the SpsD-Fn interaction.

**FIG 5:**
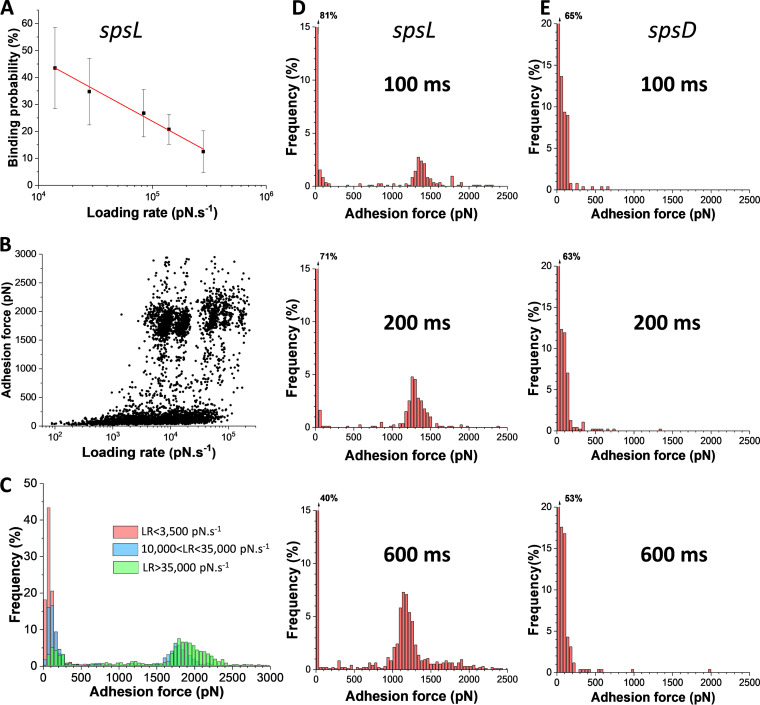
Effect of the loading rate and contact time on the SpsD-Fn interaction. (A) Variation of the probability of SpsD-Fn interactions with the LR. (B) Dynamic force spectroscopy data showing the strength of the SpsD-Fn interaction at increasing loading rates (LRs). (C) Small ranges of LRs were binned, and the force distributions were plotted as histograms, revealing that the probability of forming strong bonds increased with the LR. (D, E) Influence of the contact time on the binding probability of the SpsD-Fn (D) and SpsL-Fn (E) interactions.

To investigate the dynamics of the SpsD-Fn interaction, force curves were recorded while varying the rate at which force is applied (loading rate [LR]). Typically, the unbinding force of specific bonds increases with the LR, an observation that has been widely described by the Bell-Evans (20) and Friddle et al. (21) models. Recently, S. aureus adhesins ClfA and ClfB were shown to follow a different trend (22, 23). These proteins bind their ligand through the multistep dock, lock, and latch (DLL) mechanism involving dynamic conformational changes of the protein. Upon increasing the LR, the binding strength of ClfA and ClfB is dramatically enhanced from 100 to 250 pN to ∼1,500 pN, similar to a catch bond mechanism. We, therefore, asked whether the SpsD-Fn complex follows one of these two behaviors. The strength of the interaction (*F*) was measured at different LRs (the effective LR was estimated from the force versus time curves). The probability of forming strong bonds decreased with the LR (Fig. 5A), while the binding strength remained unchanged. We also found that the bond is weak (∼0.25 nN) at low tensile force but is dramatically enhanced (∼2 nN) by mechanical tension as observed with catch bonds (Fig. 5B and C). We hypothesize that the strong bond is activated by tensile force and that, when it is loaded quickly, the interaction time between SpsD and Fn might be too short to allow for conformational changes and optimal fitting between the active binding sequences. Supporting this idea, we found that increasing the interaction time from 100 to 600 ms increased the probability of forming strong SpsD-Fn bonds (Fig. 5C), while it had no effect on SpsL-Fn bonds (Fig. 5D).

## DISCUSSION

We have shown that the SpsD-Fn interaction is extremely strong, which, together with the recently discovered DLL binding mechanism (24, 25), represents the highest mechanical strength reported to date for a noncovalent biological interaction. As in DLL complexes, the strong SpsD-Fn interaction is activated by mechanical tension as observed with catch bonds (26).

Owing to single-molecule experiments, we are now starting to appreciate that pathogens have evolved molecular interactions that are extremely strong, enabling them to firmly attach to their host during colonization and infection. The SpsL-Fn interaction is much weaker, with a binding strength similar to that of classical receptor-ligand complexes (<0.2 nN). Interaction strengths correlate with dissociation constants, with SpsD featuring a remarkably high affinity (*K_D_* of ∼1 nM versus ∼50 nM for SpsL). These observations suggest that SpsD and SpsL have different mechanisms for binding to Fn, which is unexpected and surprising as the two adhesins share sequence similarity with S. aureus FnBPs and fulfill the same invasion function.

**FIG 6:**
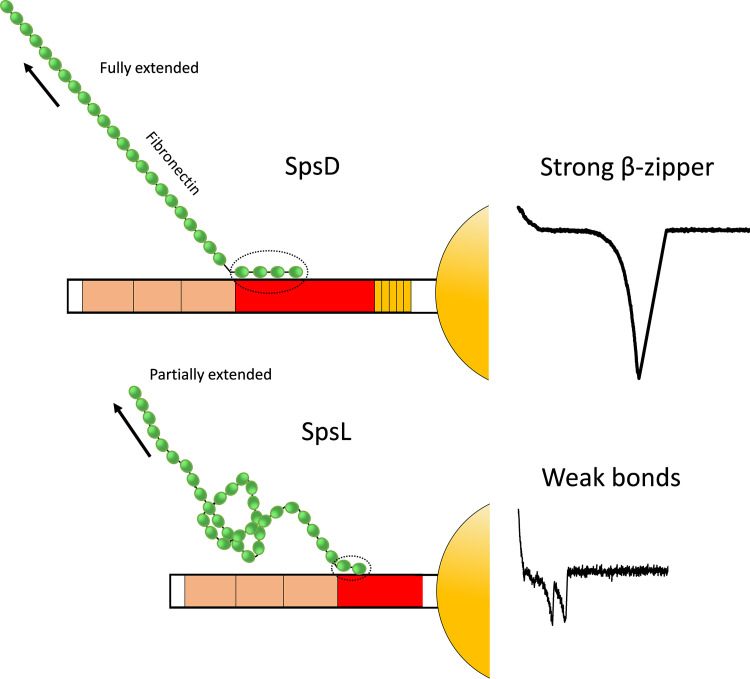
SpsD and SpsL bind Fn through distinct mechanisms. We propose a model where the mechanostability of the SpsD-Fn interaction originates from the β-sheet organization of a tandem β-zipper. The globular form of soluble Fn undergoes a conformational change to an extended form so that high-affinity RGD binding sites may become available for interaction with integrin α_5_β_1_. The extremely strong SpsD-Fn interaction would result from the simultaneous rupture of all bonds of the β-zipper. In sharp contrast, the weak SpsL-Fn interaction involves the sequential unbinding of single bonds not engaged in a β-zipper.

We speculate that the mechanostability of the SpsD-Fn interaction originates from the β-sheet organization of a tandem β-zipper (Fig. 6, top) as identified previously for FnBPs, Sfb1, and BBK32 from the pathogens Staphylococcus aureus (14), Streptococcus pyogenes (14), and Borrelia burgdorferi (27), respectively. When SpsD binds to FnI modules, its intrinsically disordered connecting region (region C) would shift into an ordered structure by forming additional β-strands along triple peptide β-sheets in the Fn molecule. We can also hypothesize that like the allosteric regulation of the Fn-α_5_β_1_ interaction by FnBPA (28), the globular form of soluble Fn undergoes a conformational change to an extended form so that ligand (integrin)-binding sites on FnIII modules are exposed and become available for interaction. This model is supported by LIBS MAbs and sequence alignments revealing that SpsD shares with FnBPA disordered epitopes that acquire an ordered structure upon Fn binding. The use of LIBS antibodies has also provided cues to identify minimal Fn-binding units in both SpsD and SpsL, and this can pave the way for designing peptide analogs with inhibitory potential on Fn interactions (18).

Moreover, the SpsD-Fn complex has a high affinity of 1 nM, in line with the work of Meenan et al. (18), who found that 6 of the 11 Fn-binding sites of FnBPA bind with dissociation constants in the nanomolar range. The strong, well-defined SpsD adhesion peaks are consistent with an unbinding model in which multiple bonds of the SpsD-Fn β-zipper rupture simultaneously and cooperatively, thus resulting in a mechanically stable complex (Fig. 6, top). Structural analysis of the FnBPA-Fn β-zipper have suggested that electrostatic interactions, hydrophobic forces, and hydrogen bonds are likely to be important for binding (14, 29). Further molecular dynamics simulations and structural analysis will greatly contribute to understanding the molecular bonds behind the extreme mechanostability of the SpsD-Fn interaction. Milles et al. (24) and Herman-Bausier and Dufrêne (25) applied this approach to the DLL interaction between the staphylococcal adhesin SdrG and fibrinogen. They discovered that the target peptide is confined in a screw-like manner in the binding pocket and that the binding strength of the complex results from numerous hydrogen bonds between the peptide backbone and the adhesin.

The interaction between SpsL and Fn involves weak bonds that rupture sequentially, indicating that they are not engaged in a strong β-zipper complex (Fig. 6, bottom). That SpsL requires much lower separation force than SpsD is likely to be due to sequence diversity, resulting in conformational differences between the adhesins. This hypothesis is supported by recent studies showing that amino acid substitutions in the repeat region of FnBPA significantly affect bond strength and influence the conformation of Fn upon binding (19, 30). Therefore, intrinsically disordered sequences from both adhesins may bind to FnI β-sheets, but SpsL would not form a β-zipper because of differences in the spacing, flexibility, and conformation of active sequences. SpsL-Fn rupture forces feature multipeaks that fit with the unfolding of single SpsL FnBRs, while these are intrinsically disordered in their native state. Perhaps FnBRs become more ordered upon Fn binding. Due to the weak SpsL-Fn interaction, Fn remains in a compact conformation as in its soluble state, and upon pulling the complex apart, Fn becomes partially extended explaining the short extensions we observed (∼100 nm) (Fig. 4E and F). Upon pulling the complex apart, Fn becomes partially extended and some FnIII modules become unfolded (26) (Fig. 6, bottom). The weak binding strength correlates with the lower level of identity/similarity of SpsL with FnBPA compared to that of SpsD, as well as with its relatively lower affinity for Fn. We propose that binding strengths (at nonequilibrium) are more appropriate than binding affinities (at equilibrium) to understand the binding mechanism of SpsD-Fn complexes. As most surface-attached bacteria are subjected to physical stress, it seems more appropriate to study their molecular bonds under force as in the present study. Also, it is known that adhesins sharing a common binding mechanism to Fn do not have similar affinity. *K_D_* values for FnBPA/FnBPB are in the range of 1 to 10 nM, while the N terminus of BBK32 is in the order of 100 nM.

The different interaction strengths of SpsD and SpsL could be of biological significance in that they may play complementary roles in invasion and dissemination. There is evidence that invasion by S. pseudintermedius involves an Fn bridge between SpsD or SpsL and the α_5_β_1_ integrin in the host cell membrane (12). We, therefore, postulate that the strong SpsD-Fn β-zipper may expose high-affinity RGD sites, as in the FnBPA β-zipper, leading to a mechanically stable bridge. Formation of SpsD-Fn nanodomains on the bacterial cell surface would favor integrin clustering and invasion. The weak SpsL-Fn interaction and lack of clustering would mediate moderate adhesion to the α_5_β_1_ integrin and favor detachment of the pathogens from the host cell surface, thus enabling the colonization of new sites.

## MATERIALS AND METHODS

### Construction of *spsD*- and *spsL*-null bacterial mutants and growth conditions.

Construction of *spsD*- and *spsL*-null mutants (*spsD* and s*psL* mutant strains, respectively) was performed as previously reported (12). All strains were grown in brain heart infusion (BHI) broth overnight at 37°C and under shaking at 200 rpm to reach their stationary phase. For AFM experiments, cells were harvested by centrifugation at 3,000 × *g* for 5 min and washed twice with phosphate-buffered saline (PBS).

### Proteins.

Bovine serum albumin (BSA) was purchased from Sigma-Aldrich. Human fibrinogen was obtained from Calbiochem (Darmstadt, Germany). Collagen type I was a gift of R. Tenni (Department of Molecular Medicine, University of Pavia, Pavia, Italy). Factor H (FH) and factor I (FI) were from Merck Millipore (Darmstadt, Germany).

### DNA manipulation and expression of recombinant proteins.

DNA manipulation, expression of SpsD_520–846_ and SpsL_538–823_, and their purification were performed as previously reported (12).

### Purification of plasma Fn.

Human Fn was purified from plasma by a combination of gelatin and arginine Sepharose affinity chromatography (31). The N-terminal fragment of Fn (N29) was isolated as reported by Zardi et al. (32).

### Antibodies.

Polyclonal antibodies against human Fn and fibrinogen were raised in mouse by routine procedures using purified antigens. Polyclonal antibodies against factor H (FH) and factor I (FI) were raised in rabbit. Rabbit antibodies against type I collagen were purchased from Sigma. The antibodies were purified by affinity chromatography on Protein A/G Sepharose columns according to the recommendations of the manufacturer (GE Healthcare). Rabbit anti-mouse or goat anti-rabbit horseradish peroxidase (HRP)-conjugated secondary antibodies were purchased from DakoCytomation (Glostrup, Denmark).

### Bacterial adhesion to immobilized Fn.

Microtiter wells were coated overnight at 4°C with 1 μg/well human Fn in 0.1 M sodium carbonate (pH 9.5). The plates were washed with PBS containing 0.5% (vol/vol) Tween 20 (PBST). To block additional protein-binding sites, the wells were treated for 1 h at 22°C with 2% (vol/vol) bovine serum albumin (BSA) in PBS. The wells were then incubated for 1 h at 37°C with 1 × 10^8^ cells of the S. pseudintermedius strain ED99 or its mutants. After being washed with PBS, adhering cells were fixed with 2.5% formaldehyde for 30 min and stained with 1% crystal violet for 1 min. Following several washings, 100 μl of 10% acetic acid were added, and absorbance at 595 nm was recorded in a plate reader (Bio-Rad). To test the inhibitory effect of soluble N29, the adhesion assay was performed as above in the presence of 5 μg N-terminal domain (N29) of Fn.

Adhesion of S. pseudintermedius ED99 and derivative mutants was also assessed on Fn-functionalized surfaces (see below) using optical microscopy in static conditions. Bacterial suspensions in PBS were incubated on Fn surfaces for 2 h at 37°C, gently rinsed with PBS, and imaged using an optical microscope Zeiss Axio Observer Z1 and a Hamamatsu camera C10600.

### Binding of extracellular matrix and plasma proteins to immobilized SpsD_520–846_ and SpsL_538–823_.

SpsD_520–846_ or SpsL_538–823_ recombinant proteins (1 μg) dissolved in 0.1 M sodium carbonate, pH 9.5, were immobilized overnight onto microtiter wells. The plates were washed with PBS containing 0.5% (vol/vol) Tween 20 (PBST). To block additional protein-binding sites, wells were treated for 1 h at 22°C with 2% (vol/vol) bovine serum albumin (BSA) in PBS and then incubated for 90 min with 1 μg Fn, fibrinogen, collagen type I, factor H, and factor I. Binding of each protein to the surface-coated bacterial proteins was determined by addition to the wells of specific antibodies (1 μg/well) to each ligand and incubation for 60 min. After several washings, wells were added and incubated for 60 min with peroxidase-conjugated rabbit anti-mouse IgG or peroxidase-conjugated goat anti-rabbit IgG (1:1,000). After washing, bound conjugated enzyme was detected incubating the wells with a chromogenic substrate (*o*-phenylenediamine dihydrochloride), and the absorbance at 490 nm was determined in a plate reader.

### Surface plasmon resonance analysis of Fn binding by SpsD and SpsL.

Surface plasmon resonance (SPR) was performed using a Biacore X100 instrument (GE Healthcare). Recombinant SpsD_520–846_ or SpsL_538–823_ were covalently immobilized on dextran matrix CM5 sensor chips in two different flow cells by using a protein solution (50 μg/ml in 50 mM sodium acetate buffer, pH 4.5) in a 1:1 dilution with *N*-hydroxysuccinimide and 1-ethyl-3-(3-dimethylaminopropyl) carbodiimide hydrochloride. The excess of active groups on the dextran matrix was blocked using 1 M ethanolamine, pH 8.5. On another flow cell, the dextran matrix was treated as described above but without any ligand to provide an uncoated reference flow cell. The running buffer was PBS containing 0.005% (vol/vol) Tween 20. A 2-fold linear dilution series of Fn in running buffer was passed over the ligand at a flow rate of 30 μl/min, and all of the sensorgrams were recorded at 22°C. Assay channel data were subtracted from reference flow cell data. The response units at the steady state were plotted as a function of Fn concentration and fitted to the Langmuir equation to yield the *K_D_* values.

### Binding of MAbs to SpsD_520–846_ and SpsL_538–823_ recombinant proteins.

MAbs directed toward full-length FnBPA were raised as previously described (18, 33). Microtiter wells coated with SpsD_520–846_ and SpsL_538–823_ (10 μg/ml) were preincubated with PBS alone or with Fn (5 μg/ml) in PBS. After washing with PBST, 100 μl of the MAbs 1F9, 5G3, 6B7, or 7D4 (10 μg/ml) was added to the wells before incubation at 22°C for 1 h. Bound MAbs were detected by incubation with a 1:1,000 dilution of HRP-conjugated rabbit anti-mouse polyclonal antibodies. The binding of the secondary antibody was quantified by adding the substrate *o*-phenylenediamine dihydrochloride and measuring the resulting absorbance at 490 nm in a plate reader.

### Circular dichroism spectroscopy.

Far-UV (185 to 260 nm) CD measurement was performed at a 10 μM protein concentration in a 0.1-cm path length quartz cuvette using a Jasco J-1500 spectropolarimeter (Jasco, Easton, MD, USA). The results are expressed as the mean residue ellipticity assuming a mean residue weight of 110. All measurements were performed in 20 mM phosphate buffer, pH 7.4, at 25°C. Ten scans were averaged for each spectrum, and the contribution from the buffer was subtracted in each case. Quantification of secondary structural components was performed using the deconvolution programs CONTIN, CDSSTR, and SELCON3, and the values reported are an average of results obtained.

### Functionalization of substrates and cantilevers with Fn.

Gold-coated glass coverslips and gold cantilevers (OMCL-TR400PB-1; Olympus Ltd., Tokyo, Japan; nominal spring constant of ∼0.02 N · m^−1^) were immersed overnight in an ethanol solution containing 1 mM 10% 16-mercaptododecahexanoic acid/90% 1-mercapto-1-undecanol (Sigma), rinsed with ethanol and dried with N2. Substrates and cantilevers were then immersed for 30 min into a solution containing 10 mg · ml^−1^ *N*-hydroxysuccinimide (NHS) and 25 mg · ml^−1^ 1-ethyl-3-(3- dimethylaminopropyl)-carbodiimide (EDC) (Sigma) and rinsed with Ultrapure water (ELGA LabWater). Finally, they were incubated with 0.1 mg · ml^−1^ of Fn for 1 h, rinsed further with PBS buffer, and then immediately used without dewetting.

### Single-cell force spectroscopy.

Colloidal probes were prepared as described earlier (34). The nominal spring constant of cantilevers was determined by the thermal noise method, giving an average value of ∼0.08 N/m. Briefly, 50 μl of a suspension of ca. 1 × 10^6^ cells was transferred into a glass petri dish containing Fn-coated substrates on the other corner, the whole being immersed in PBS. The colloidal probe was brought into contact with a bacterium, which is first caught through electrostatic interactions with polydopamine. The cell probe was then positioned over Fn substrates without dewetting. Cell probes were used to measure interaction forces on Fn surfaces at room temperature by recording multiple force curves (16 by 16) on different spots, a maximum applied force of 250 pN, and approach and retraction speeds of 1,000 nm s^−1^ and a contact time of 100 ms. Data were analyzed with the data processing software from JPK Instruments (Berlin, Germany). Adhesion force and rupture distance histograms were obtained by calculating the adhesion force and rupture distance of the last peak for each curve. At least 10 cells of each strain from 3 independent cultures were probed.

### Single-molecule force spectroscopy.

Cantilevers (*k*, ∼0.02 N/m) were prepared as described above, and bacteria were immobilized on polystyrene substrates. Measurements were performed at room temperature in PBS buffer with a NanoWizard 4 atomic force microscope (JPK Instruments). Adhesion maps were obtained by recording 32 by 32 force-distance curves on areas of 500 by 500 nm^2^ with an applied force of 250 pN, a constant approach and retraction speed of 1,000 nm · s^−1^, and a contact time of 100 ms. For some experiments, the contact time was increased from 100 ms to 200 and 600 ms. For loading rate experiments, arrays of 32 by 32 force curves were recorded on 500-nm by 500-nm areas at increasing retraction speeds as follows: 0.5, 1, 3, 5, and 10 μm · s^−1^. Single-molecule data were processed and analyzed the same as for single-cell experiments. Adhesion force and rupture distance histograms were obtained by calculating the adhesion force and rupture distance of the last peak for each curve. At least 10 cells of each strain from 3 independent cultures were probed.
