# Supplementary material for: The Molecular Complex between Staphylococcal Adhesin SpsD and Fibronectin Sustains Mechanical Forces in the Nanonewton Range
Source: mBio. 2020 Jul 7;11(4):e00371-20. doi: 10.1128/mBio.00371-20 (PMC7343985; doi:10.1128/mBio.00371-20)
Supplement: FIG S1 [file mBio.00371-20-sf001.docx]

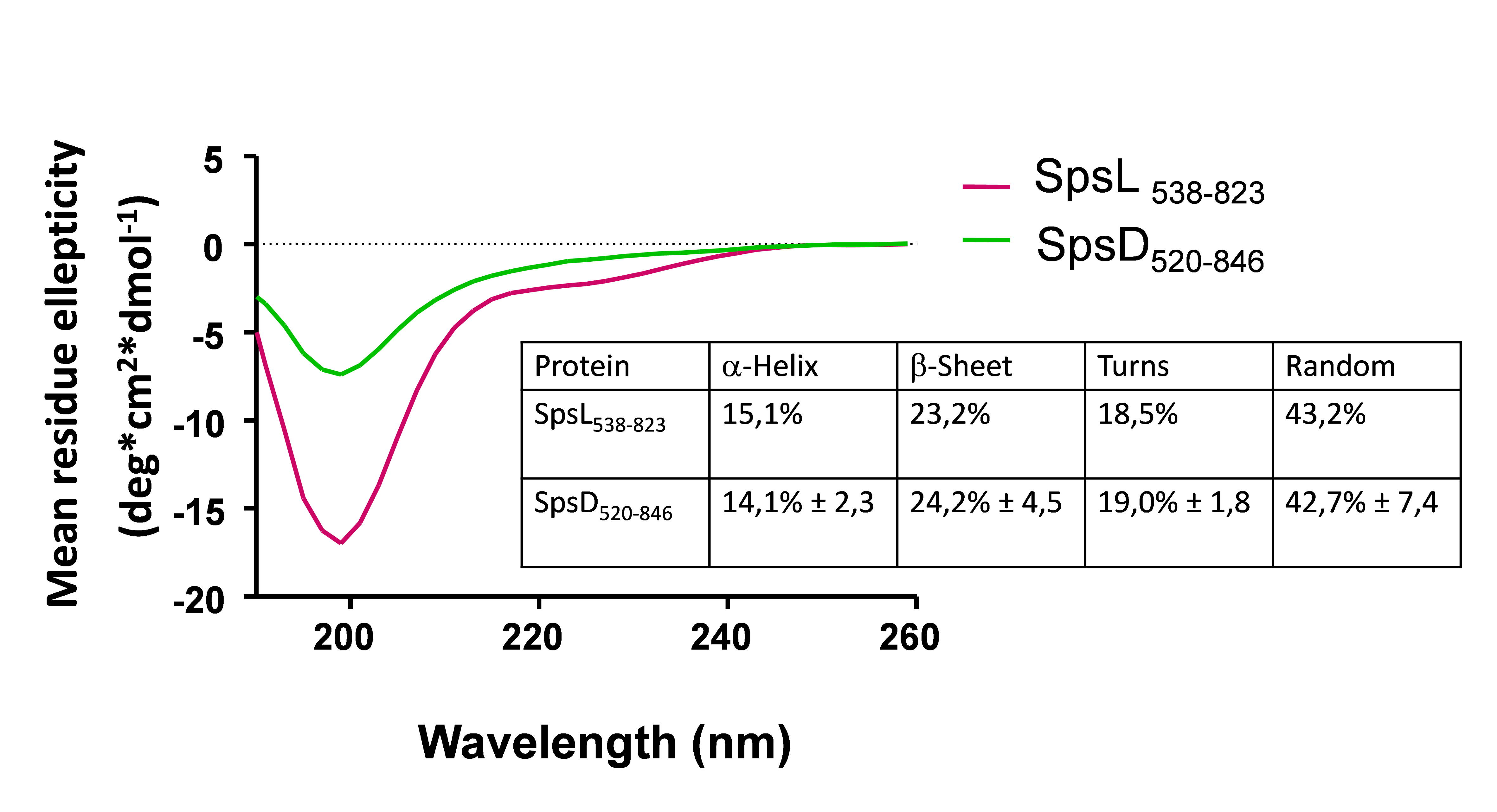


**Figure S1. Spectroscopic analysis of SpsL538-823 and SpsD520-846 domains**. Far-UV CD spectra were recorded at 10 μM concentration for both proteins in 20 mM phosphate buffer, pH 7.0. The predicted secondary structure composition of each protein is reported in the insert. The spectra are the average of ten scans and corrected for buffer blank.
