# Supplementary material for: The Molecular Complex between Staphylococcal Adhesin SpsD and Fibronectin Sustains Mechanical Forces in the Nanonewton Range
Source: mBio. 2020 Jul 7;11(4):e00371-20. doi: 10.1128/mBio.00371-20 (PMC7343985; doi:10.1128/mBio.00371-20)
Supplement: FIG S3 [file mBio.00371-20-sf003.docx]

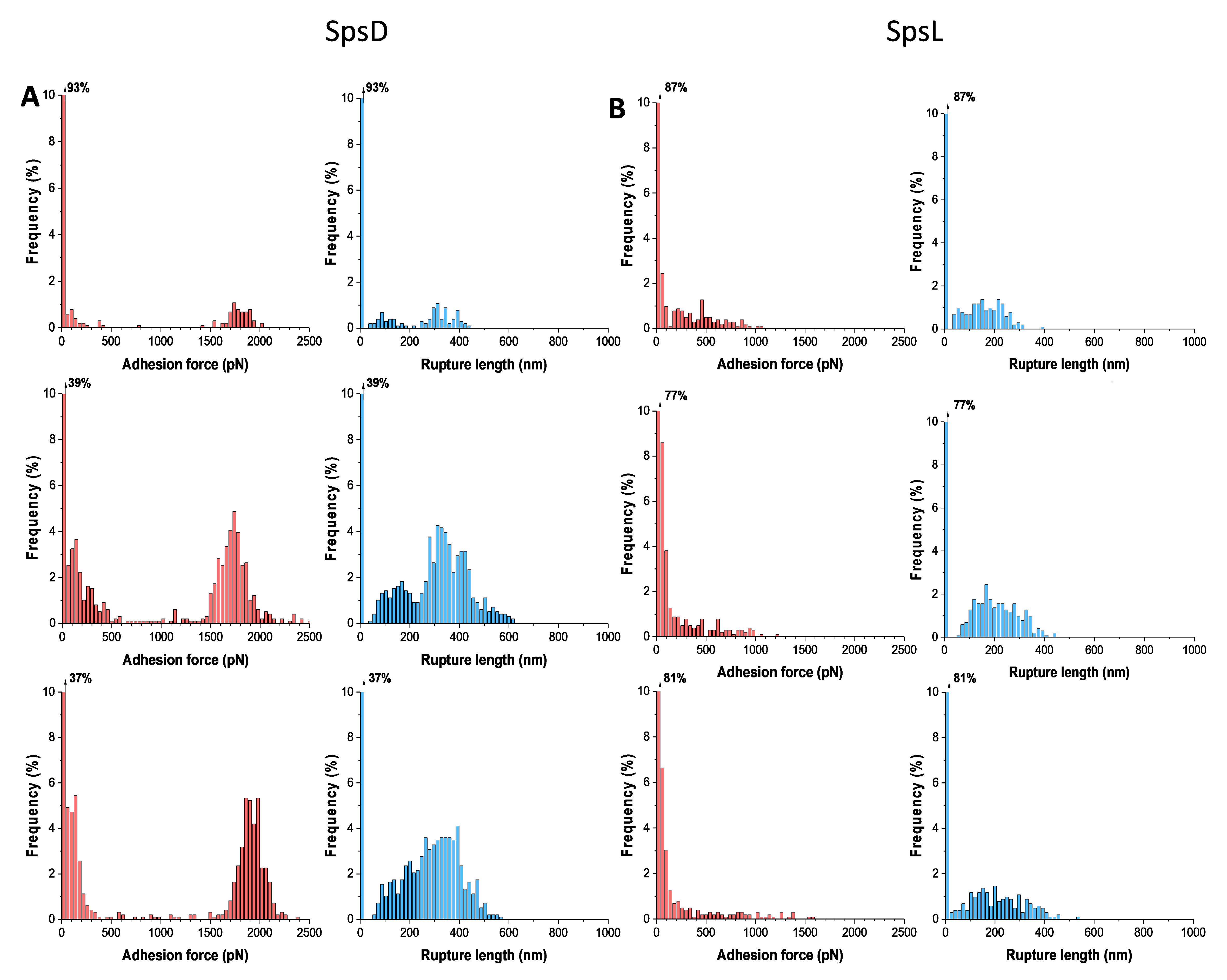


**Figure S3. Single-molecule force spectroscopy of SpsD/SpsL-Fn interactions.** Maximum adhesion force (left) and rupture length (right) histograms obtained by recording force-distance curves in PBS between Fn functionalized tips and three additional *S. pseudointermedius* *spsL* (*A*) and *spsD* (*B*) cells.
